# Current Status, Challenges, and Policy Recommendations of China’s Marine Monitoring Systems for Coastal Persistent Organic Pollution Based on Experts’ Questionnaire Analysis

**DOI:** 10.3390/ijerph16173083

**Published:** 2019-08-25

**Authors:** Wenlu Zhao, Huorong Chen, Jun Wang, Mingyu Zhang, Kai Chen, Yali Guo, Hongwei Ke, Wenyi Huang, Lihua Liu, Shengyun Yang, Minggang Cai

**Affiliations:** 1 School of Environmental Science and Engineering, Zhejiang Gongshang University, Hangzhou 310018, China; 2 Fujian Provincial Key Laboratory for Coastal Ecology and Environmental Studies, Xiamen University, Xiamen 361102, China; 3 College of Ocean and Earth Science, Xiamen University, Xiamen 361102, China; 4 Monitoring Center of Marine Environment and Fisheries Resources of Fujian, Fuzhou 350003, China; 5 Department of Biological Technology, Xiamen Ocean Vocational College, Xiamen 361102, China; 6 Coastal and Ocean Management Institute, Xiamen University, Xiamen 361102, China; 7 East Sea Marine Environmental Investigating and Surveying Center, Ministry of Natural Resources, Shanghai 200137, China; 8 Guangzhou Institute of Energy Conversion, Chinese Academy of Sciences, Guangzhou 510640, China

**Keywords:** persistent organic pollutants, marine monitoring, public participation, policy, China

## Abstract

Persistent organic pollutants (POPs) monitoring and management in typical semi-enclosed bays is a major global environmental issue. This study concentrated on a questionnaire survey and analysis of marine environmental management and monitoring departments at all levels in China, and proposed suggestions on the construction and improvement of POPs monitoring and management system. Results show that POPs are initially involved in China’s current marine environmental monitoring system, and the monitoring strength and capability still need to be continuously improved, mainly in the recognition, funding input, relevant standards, monitoring, and evaluation technical regulations of marine environmental POPs monitoring. Therefore, in order to gradually improve the monitoring and management system of China’s offshore marine environment POPs, this study suggests starting from four directions: (1) Building POPs monitoring system of a marine ecological environment, and strengthening POPs monitoring in different environmental media; (2) strengthening land-based POPs emission and the related human activities’ intensity survey, and establishing a POPs information sharing database; (3) optimizing POPs monitoring technology in the marine environment, and improving POPs supervision and management technical support system; and (4) participating in regional and international marine environment POPs monitoring and evaluation projects, and strengthening the construction of talent teams.

## 1. Introduction

Persistent organic pollutants (POPs) are chemicals with semi-volatile, persistent in the environment, bio-accumulative, and highly toxic properties, which can migrate over long distances through various environmental media, such as air, water, soil, and sediments, and will cause serious harm to human health and the environment [1]. The concern about POPs started in 1962 when Rachel Carson drew attention to organochlorine pesticides in her book *Silent Spring* [2]. From then on, with environmental hazards disclosed by pollution incidents and research papers, the international society gradually began to take a series of control measures to regulate and control the production and use of toxic chemicals [3,4,5]. In May 2001, representatives of 127 countries and regions, including China, signed “The Stockholm Convention on Persistent Organic Pollutants” in Stockholm, which aims to reduce the emission of POPs to protect environment and human health from harm. At first, the convention listed 12 priority control POPs, which were considered as “legacy” POPs, and then added 16 new POPs during 2009 to 2017 [6]. Although a series of international, regional, and national actions have been carried out to eliminate or reduce the production and use of POPs [7,8,9,10], they still migrate and redistribute in different environmental media. It is necessary to change the traditional thinking about POPs pollution, and to strengthen the attention and restriction implemented to them, especially for “new” POPs (contaminants of emerging concern), such as pharmaceutical and personal care products (PPCPs), disinfection by-products, surfactants, plasticizers and industrial additives, and their degradation products [11,12,13,14], as they spread to the environment after being used in commerce, industry, and daily life and are often out of the monitoring scope [3,15]. Polycyclic aromatic hydrocarbons (PAHs) are another class of POPs with ongoing emissions and serious harm to human health, and they have been added to the list of POPs and regulated under the UNECE Convention on Long-Range Transboundary Air Pollution (LRTAP) and the OSPAR Convention [16,17]. Obviously, as we know more about POPs and synthesize more new chemicals, the categories of controlled and eliminated POPs will continue to increase. 

Marine environment monitoring is an important part of marine environmental protection, and is also a basic method for governments to supervise and manage marine environments [18]. The marine environmental monitoring network of China was developed in 1984, which has set up a solid foundation for marine environmental assessment and resource management. The monitoring has shown that the marine environment is contaminated mostly in estuaries, bays, and enclosed water bodies adjacent to large cities. The principal contaminants are oil, nutrients, and organic matter, and mainly come from various land-based pollution sources [19]. In recent years, several researches have focused on the technology development [20], network optimization [21,22], and information management [23] of marine environmental monitoring, and have applied them gradually. At the same time, the Chinese government has proposed several macrolevel suggestions on environmental management, such as establishing a land–marine coordination mechanism for environmental monitoring. However, with the coastal pollution in China being more obvious, especially for POPs [15,24,25,26], it is of great importance to further study how to develop a robust monitoring system for POPs in terrestrial coastal offshore areas.

So far, through the global monitoring plan, POPs in air, water, and human samples have been measured under the Stockholm Convention to better know their emissions, long-range transport, and exposure status [27]. China has begun to actively participate in international cooperation since the 1990s and has issued a series of POPs-related laws and regulations [3,28,29]. Under this supervision system, China’s marine environment monitoring can be divided into five main tasks, including marine ecological monitoring, marine supervised monitoring, public welfare service monitoring, marine ecological risk monitoring, and pilot monitoring of the carrying capacity of marine resources and environments (according to State Oceanic Administration “National Marine Environment Monitoring Task 2014”). Among them, the numbers of projects involving POPs as monitoring indicators are still scarce, mainly focusing on marine sediments, land-based pollutants discharged into the sea, red tide, dangerous chemical pollution, marine aquaculture areas, marine dumping areas, marine oil exploration, and development areas, etc. 

On the basis of a lot of monitoring and research on POPs pollution levels in various environmental media, some challenges and threats to the implementation efficiency and future development of international conventions, as well as to the monitoring and management of POPs by governments, still exist. Based on the current status of POPs monitoring in China’s typical offshore environment (South China Sea, Fujian Province, Shandong Province, and Hainan Province), this study carried out reviews on the development status of marine POPs monitoring in China. Meanwhile, we also analyzed the awareness and capacity of marine environment POPs monitoring based on experts’ questionnaire surveys. At last, existing problems are identified, and relative development measures and suggestions are given in order to provide valuable scientific and management suggestions for the improvement of the marine POPs monitoring system.

## 2. The Development of Marine POPs Management and Monitoring in China

### 2.1. Environmental Policy and Legislation of Marine POPs in China

In the late 1970s or early 1980s, the Chinese government started the management and legislation of POPs, which is also when developed countries banned the use of highly toxic pesticides, and the public and scientists started to become concerned about their influence on humans and environments [30,31,32]. In 1979, the National People’s Congress passed the Environmental Protection Act of the People’s Republic of China (trial edition), which mentioned “popularizing the pesticides with high efficiency, low toxicity and persistence”. The Law of Marine Environment Protection was established in 1982, regulating the use of chemical pesticides according to a national safety standard. In 1983, China State Council started to ban the production and usage of several highly toxic pesticides, such as Dichlorodiphenyltrichloroethane (DDT) and Hexachlorocyclohexane (HCH) [4]. Although the Chinese government has a strong expectation on banning toxic pesticides, it lacks effective supervision and executive force [5].

In the next 30 years, several acts related to POPs have been approved or revised by the National People’s Congress of China, including the Law of Marine Environment Protection (1982, 1999, 2013, 2016, 2017), the Law of Environmental Protection (1989, 2014), the Law of Prevention and Control of Water Pollution (1984, 1996, 2008, 2017), the Law of Prevention and Control of Environmental Pollution by Solid Waste (1995, 2016), the Law of Prevention and Control of Atmospheric Pollution (1987, 1995, 2000, 2015, 2018), the Law of Appraising of Environment Impacts (2002, 2016, 2018), the Law of Quality and Safety of Agriculture Products (2006; 2018), the Law of Prevention and Control of Occupational Disease (2001, 2011, 2016, 2017, 2018), and the Law of Safety in Production (2002, 2009, 2014). Always listed in the category of pesticides or hazardous chemicals, POPs were regulated not only by the laws but also by regulations and ordinances, which provide direct instructions in marine POPs management, such as the Regulations of Chemicals Control (1995, 2011), the Regulations of Safety for Hazardous Chemicals (2002, 2011, 2013), the Regulations of Pesticide Labels and Instructions Management (2007), and the Regulations of Recycling and Treatment of Waste Electrical and Electronic Products (2009). With the reference of more than 20 standards, a complete legal framework is under establishment, which covers acts, regulations, ordinances, and standards [3,33].

At the same time, China signed several international conventions related to POPs management, such as London Guidelines for the Exchange of Information on Chemicals in International Trade (1989), Convention on safety in use of chemicals at work (1990), Basel Convention on the Control of Transboundary Movements of Hazardous Wastes and Their Disposal (1989), Rotterdam Convention on the Prior Informed Consent Procedure for Certain Hazardous Chemicals and Pesticides in International Trade (1998), Stockholm Convention on Persistent Organic Pollutants (2011), and International Code of Conduct on the Distribution and Use of Pesticides (2003). Through this extensive international cooperation, there has been significant improvement in policy legislation, personnel quality, analytical equipment, and data prediction, strengthening the power of marine POPs management and monitoring as well [3,34].

### 2.2. Monitoring Status of Marine POPs in China

This study collected monitoring projects that explicitly involve POPs in China’s marine environment monitoring during the 12th Five-Year Plan period. The South China Sea, Hainan Province, Shandong Province, and Fujian Provinces represented the POPs monitoring involved in various marine regions and individual provinces. 

Through the comparative analysis of marine environment monitoring in different regions, the results show that, in general, the marine environment monitoring of all provinces in China has continuously strengthened their capacity building. Currently, the coastal POPs monitoring has analyzed samples from mussels, sediments, and seawaters, and in some special situations, such as engineering construction, dumping and sewage draining, red tide, offshore petroleum exploration, and hazardous chemical monitoring [35,36,37,38]. The hazardous chemical monitoring currently lacks detailed operating regulations; the target chemicals are selected according to their influence on water, sediments, and the biota, and the monitoring time and frequency are determined by their discharge load and the diffusion range [38,39].

For the monitoring indicators, the differences in projects involving POPs monitoring are generally small, mainly concentrated in sediments and the monitoring of HCHs, DDTs, and PCBs in living organisms. The main problems are the lack of monitoring of seawater itself and the need to expand monitoring to other types of POPs. At the same time, the monitoring of each province has its own characteristics, and there are certain differences. For example, Shandong Province has continuously strengthened the monitoring of POPs. From 2013 to 2015, it increased the selection of POPs (organophosphorus pesticides, organochlorine pesticides, PAHs, PCBs) in the rivers and other environmental endocrine disruptors indicators; in 2014, the [35,36,37] seawater aquaculture area increased the selection of aquaculture water in PAHs, DDTs, PCBs, antibiotics, and other indicators; in 2015, it increased seawater quality in HCHs, DDTs, PCBs, and PAHs monitoring, etc. In addition to specifying the type and information of sewage discharges in specific sewage outlets, the sea areas in Fujian Province have also identified different monitoring items according to the sewage discharge characteristics of different sewage outlets. For the sewage outlets where POPs pollutants are necessary, the corresponding monitoring indicators have been clarified (Table 1 and Table 2).

To solve POPs pollution in coastal-offshore environments effectively, it is necessary to better understand the current status of POPs monitoring in marine environments. With optimization of the monitoring plans, methods, and standards, the efficiency of marine POPs monitoring is expected to improve.

## 3. Awareness and Capacity of Marine Environment POPs Monitoring in China

### 3.1. Public Participation

In order to explore perceived barriers to implementing POPs monitoring in China’s marine environment, we investigated the perceptions of stakeholders who are represented by key groups of first-line staff and experts in marine environmental monitoring and research in China. The 99 interviewees work at representative marine environmental monitoring centers, universities, and other oceanographic institutions from coastal provinces of China (supplemental Table S1). With the focus on their perception, awareness, attitude, and willingness to pay for POPs monitoring and management, we designed and conducted the “Questionnaire on POPs monitoring in the marine environment” in this study (in Supplementary Material). 

The questionnaire incorporated single-choice, multiple-choice, and subjective questions. It consisted of six major themes: (1) Overview of participation in marine monitoring, (2) awareness of POPs, (3) POPs monitoring operation in the marine environment, (4) the ability for POPs analyzing, data processing, and sharing, (5) training mechanisms to monitoring technicians, and (6) quality control and assurance. We also collected opinions and suggestions on the improvement of marine POPs environmental monitoring and management from these interviewees. In total, 98% of the total responses were valid, which met the requirements of general survey statistical standards. All the statistical analysis was operated using Office^®^ Excel 2016 (Microsoft, Redmond, WA, U.S.).

### 3.2. The Overall Situation of Marine Environmental Monitoring in China 

Respondents also expressed their concerned monitoring objects and areas during their regular monitoring. In total, 85.7% of the respondents indicated that it should focus on the on-site, real-time, and online monitoring of key drainage, ecologically sensitive, and red-tide areas. Moreover, the respondents also thought that it was significant to determine the list of China’s environmental priority control pollutants and carry out early-warning monitoring of red tides, oil spills, ecological diseases, and new pollutants. 

However, existing problems still affect the improvement of China’s marine environmental monitoring technology. Over 85% of respondents considered that the discrepancy between monitoring technical resource allocation and capacity distribution is the main reason. Other factors included the incompatibility between the existing behindhand monitoring standards and advanced techniques, and the weak foundation of marine monitoring institutions, which cannot catch up with advanced monitoring technologies well.

### 3.3. Cognition on POPs and Relevant Training Mechanisms

The survey results showed that many frontline monitors are still not fully aware of the properties, types, and sources of POPs. More than half of the respondents (55.1%) thought they had a “general understanding” of POPs, while 12.2% of them thought they had “no understanding” of POPs. There was also cognitive polarization on the status of global POPs monitoring. Although a large proportion of frontline monitors basically understood the current international development status, there was still about one-third of them that did not care about it.

The cognition of frontline monitors on POPs was strongly related to the training mechanisms. Most of the respondents (95.8%) indicated that they had not or occasionally received related training in their work institution, while only 4.2% indicated that they received regular related training. For the international cooperation and exchange involving POPs in marine environmental monitoring, only 9.8% of the respondents indicated that they had participated in an international symposium, special lectures, or technical trainings on marine environmental monitoring and assessment, or had visited relevant foreign agencies. 

### 3.4. Current Status and Problems of POPs Monitoring and Analysis in the Marine Environment

Almost all the respondents, 91.8% indicated it is necessary to regularly investigate the priority control POPs in international convention at different recognized frequencies for different environmental media. In rapidly developing China, both a decreasing trend for some traditional POPs and/or increasing trend for some emerging POPs has been observed in the marine environment, such as PCDD/Fs, HCB, and PFOs [40]. However, 89.8% of the respondents indicated that POPs monitoring operations were difficult to some extent. The operational difficulties mainly included two factors, which were an unclear pollution status and monitoring indicator analysis. Meanwhile, an insufficient quantity and technical force of monitors makes it difficult for monitoring agencies to match POPs sampling, which aggravates the difficulties of sample collection and analysis. A recent review pointed out that there are more than 70 dioxin analysis labs equipped with high-resolution mass spectrum in China, and 25 of them performed well in the global test organized by United Nations Environment Programme (UNEP) for POPs analysis quality [34]. However, considering the large area and unaveraged development in China, it is difficult to guarantee advanced equipment, financial support, and human resources for all coastal monitoring departments. 

There were also problems of backward technology in monitoring data processing, analysis, and management. Over half of the respondents considered that the reliability of existing POPs monitoring data was “general" and three main reasons identified as being responsible were the representation of samples, analytical equipment, and evaluation methods and standards. Due to POPs in the environment usually being found at low concentrations (e.g., ng·L^−1^ and pg·L^−1^), enrichment pretreatment is required for as chromatography–mass spectrometry (GC/MS) qualitative and quantitative analysis [41]. However, only GC analysis is mentioned in the current POPs analysis standard, which can hardly meet the requirement of the POPs global monitoring project launched by UNEP. About 90.4% of the respondents indicated that there are still different levels of incomprehension or unclear technical details about the current marine environmental monitoring technical regulation. Also, half of them mentioned their institutions did not respond positively to joining monitoring projects due to the lack of essential instruments and operational training. The current evaluation of POPs pollution in China’s marine environment is still mostly in the qualitative analysis phase. Direct judgements of the sources and contributions of pollution are unavailable, and there is also no mature method to the ecological toxicology caused by POPs [41]. Furthermore, China has not established a POPs concentration benchmark in the existing environmental monitoring standard system that meets the characteristics of China’s marine environment. This will result in the standard parameters not fitting the actual situation, as well as the problem of too few POPs monitoring indicators being covered by the standard and the content not being updated in time. Compared to developed countries, where the monitoring technologies of toxaphene, chlordecone, and PBDEs have been popularized, China is lacking a standard monitoring approach [42].

### 3.5. Capacity of Quality Control and Assurance in POPs Monitoring

For the quality control in POPs monitoring and analysis, 81.6% of respondents considered sample preparation and analysis as the most important, 67.4% of respondents considered sample collection as the most important, and about one third considered that field testing, sample storage, and transportation (34.7%) were also important. Respondents generally agreed that the quality control and assurance system in POPs monitoring agencies in China was developing rapidly. It was also necessary to strengthen capacity building, improve monitors’ professional operational level and laboratory conditions to meet the needs of POPs analysis and testing, and strengthen the management of data quality assurance systems.

## 4. Suggestions of Marine Environment POPs Monitoring and Management in China 

### 4.1. Build up POPs Monitoring System of Marine Ecological Environment and Strengthen POPs Monitoring in Different Marine Environmental Media

POPs monitoring systems of marine ecological environments can be improved, and relevant universities and research institutions should join the systems. At present, marine environment POPs monitoring in China does not meet the needs of POPs research and management in terms of monitoring indicators, data accumulation degree, and monitoring environmental media [43]. Therefore, it is still necessary to build a comprehensive POPs monitoring system of the marine ecological environment, and formulate and implement medium- and long-term POPs monitoring plans. Full use should be made of existing marine environmental monitoring networks and detection institutions, which will strengthen POPs monitoring concentration and trends in different marine environmental media. It is equally important to give full play to the role of universities and scientific research institutions [44]. 

POPs monitoring indicators of marine atmosphere and dry and wet deposition monitoring projects should be increased. Because of the long-distance migration ability of POPs, it is suggested that the pollution transmission characteristics of atmospheric POPs need to be understood and the construction of atmospheric POPs monitoring network and long-term monitoring in key sea areas should be strengthened, including key sewage discharge areas, eco-sensitive areas, harmful algal bloom monitoring areas, and nature reserves, which is for the assessment of sources, emissions, and risks of offshore POPs [45,46].

Long-term monitoring and data accumulation of POPs in typical sea areas should be carried out. POPs in some water bodies, sediments, and organisms have been monitored in marine environment, at present. However, there are still some problems, such as interannual differences and unclear or inconsistent indicators, which means that it is difficult to accumulate and compare data effectively. POPs monitoring needs to be more rapid and timely and requires long-term observation of its changes, because of the complexity, timeliness, and multi-source nature of the water environment [47]. Therefore, it is recommended that to realize the traceability and warning mechanism of POPs in coastal marine environments, long-term POPs monitoring and data accumulation, optimal water, sediment, and biological monitoring networks, and observation of the long-term trends of POPs pollution in key sea areas need to be achieved [34].

Further clarification of POPs monitoring indicators, monitoring time, and tracking frequency is needed. In monitoring projects, such as a marine oil spill emergency, follow-up monitoring, and dangerous chemical leakage monitoring, we suggest further defining of the monitoring indicators, monitoring time, and tracking frequency, according to the historical and actual situation of the oil spill and chemical transportation in various regions. The monitoring contents and frequency of POPs should be consistent as far as possible for each monitoring item. Setting up the monitoring stations scientifically and reasonably avoids repeated work.

### 4.2. Strengthen Investigations of Land-Based POPs Emission and Related Human Activity Intensity, and Establish POPs Information Sharing Database

Combined with human activity factors, POPs monitoring indicators were added. POPs contamination in offshore marine environments is closely related to the discharge of land-based pollutants, the intensity of human activities, and land use [48]. Now, the monitoring indicators of POPs in China’s marine ecological environment are still far from meeting the priority of POPs control in China’s implementation of international conventions. In order to extend POPs monitoring from the terrestrial environment to the coastal-offshore environment, it is very sensible and reasonable to add more POPs monitoring indicators, such as PBDEs, dioxin, and other new pollutants, which is based on new POPs requirements under the Stockholm Convention.

POPs monitoring in important areas, such as sea entrances and sewage outlets, requires strengthening and the establishment of a database of POPs for optimal pollutant control in coastal regions. In future marine ecological environment POPs monitoring, investigation of human activities should be increased appropriately, which relates to POPs source. Monitoring stations add to corresponding POPs indicators, such as river estuaries and land-based sewage outlets. According to the main types of POPs in China, relevant organizations should screen and investigate key enterprises, emphasis industries, and key fields, where the situation of production, circulation, use, import, export, inventory, waste, and emission should be sorted out. Based on this, a database of POPs for optimal pollutant control in coastal regions should be established. Thus, we will have a land–sea integrated POPs control and management mechanism, which makes the sources and intensification of POPs in coastal-zone offshore marine environments clearer, and the control and reduction of POPs more targeted. Meanwhile, the database also provides the basis for the formulation of control policies and an evaluation of actions to reduce POPs.

A sharing mechanism of POPs data and information needs to be developed, as well as an early warning system of POPs in marine environments. It is very essential that this sharing mechanism of POPs data and information is developed by synthesizing the geography, population, economy, and ecological environment of coastal-zone offshore marine environments. The early warning system of POPs needs to be established in key sea areas, high risk areas of pollution, key protected areas, and environmentally sensitive areas. We should carry out the assessment and prediction of POPs in the marine environment, and issue POPs warning signals, warning schemes, and corresponding emergency countermeasures in time. That will be based on environmental data, for example, POPs monitoring data, basic environmental attributed data (land use, ecosystem structure, etc.), and pollution source data [49].

### 4.3. Optimize the POPs Monitoring Technology, and Improve the Technical Support System of POPs Supervision and Management in the Marine Environment

Special research and development projects should be established, improving POPs monitoring technology in the offshore environment. The investment in advanced analytical instruments should be increased in the process of development, research, and application. It is needful to strengthen the internationalization of monitoring standards and technologies. Monitoring institutions also promote and apply practical and effective POPs monitoring methods rapidly, such as passive sampling technology, monitoring methods, and so on.

The technical support system of POPs supervision and management should also be improved. The accuracy of POPs data is the lifeline of its detection and analysis, and a complete and standardized quality control system is the guarantee of data accuracy. Therefore, it is essential to establish a standardized and practical monitoring technology quality control system to meet the needs of the international monitoring network. It is equally important to accelerate the revision of relevant marine environmental quality standards, and improve the technical support system of POPs supervision and management (technical specifications, methodological standards, QA/QC, etc.) [50,51].

### 4.4. Participate in POPs Monitoring and Evaluation Projects of Regional and International Marine Environment, and Strengthen the Construction of the Talent Team

Participation should be moderate and effectively lead cooperative international research in POPs monitoring. POPs are global pollutants with the characteristics of cross-border migration. The Stockholm Convention requires parties to actively conduct research related to long-distance migration of POPs. However, there are no related regional or international monitoring and evaluation projects in regard to POPs in the marine environment now. Therefore, it is suggested that under the organization of the relevant national departments, we should moderately participate in the POPs monitoring plan in the global offshore environment. At the same time, the units should be strengthened to guide those participating in the international POPs monitoring plan convened by foreign governments or organizations. We should also standardize the use of monitoring data, strengthen international cooperation and exchange, and shorten the gap with developed countries in monitoring levels [52].

POPs have a long monitoring cycle and many projects because of its particularity. The state needs to develop a POPs monitoring demonstration zone in areas that have a strong monitoring ability and serious pollution, like mariculture zones, marine engineering construction project areas, etc., in developed regions of China. Setting up a point-to-zone POPs monitoring network that covers the national offshore marine environment by strengthening regional linkages should be a priority. Finally, China should also carry out a training and exchange mechanism on POPs content and monitoring technology, and strengthen the construction of monitoring personnel for POPs.

## 5. Conclusions

This study presented insights into the current status, challenges, and policy recommendations of China’s marine monitoring systems for coastal POPs from the perspective of frontline monitors and experts. In general, the marine environment monitoring of all provinces in China has continuously strengthened their capacity building. However, there were still existing problems that affect the improvement of China’s marine environmental monitoring technology, including the discrepancy of monitoring technical resource allocation and power distribution, the incompatibility between the existing backward monitoring standards and advanced techniques, and the weak foundation of marine environmental monitoring institutions, which cannot learn and spread advanced monitoring technologies well. It is also necessary to strengthen capacity building, improve monitors’ professional operational level and laboratory conditions to meet the needs of POPs analysis and testing, and strengthen the management of data quality assurance systems. Finally, the study also proposed suggestions on the construction and improvement of POPs monitoring and management systems, which is valuable for future management applications.

## Figures and Tables

**Table 1:** Marine monitoring items including persistent organic pollutants (POPs) in the South China Sea during “the 12th Five-Year” Plan of China.

Area	Year	Monitoring Projects and Indicators	Monitoring Frequency and Time	Remarks
South China Sea	2010–2011	Inshore mussel: HCHs, DDTs, PCBs	Once/year during August–October	
		Sediment: HCHs, DDTs, PCBs	Once/year in August	PAHs monitoring in the key gulf
	2012–2015	Sediment: PCBs, *HCHs*, *DDTs*	Once/year in August	
	2011–2012	Seawater: *DDTs*, *HCHs*	Twice/year in May and August; Three times/year for key coastal areas	

	2010	Water and sediments: *DDTs*, *PCBs* in marine dumping site	Once/season	
	2014–2015	Sediments: *DDTs*, *PCBs*, *HCHs* in marine dumping site	Monitoring time shall be determined according to the intensity and frequency of dumping activities as well as the number of users in the dumping area	
	2010	Water and sediments: DDTs, PCBs in demonstration area of marine engineering construction project	Once/2 months, Add irregular examination according to the situation	
	2010–2013	Sediments and bio-quality: DDTs, PCBs, antibiotics in mariculture zone	Once/year in August	
	2010–2011	Sewage toxic and hazardous substances in key sewage outlets into the sea: choose 3–5 kinds of substances prohibited in international conventions; General sewage outlets: *choose 1–2 kinds of characteristic pollutant*;	Four times/year on a seasonal basis (March, May, August, October)	Determine monitoring items based on the main categories of seawater discharge
		Adjacent seawater, sediments: PAHs, OCPs, PCBs, phthalate, Bisphenol A, Nonylphenol and octylphenol.	Once/year in May	
	2012–2013	Industrial sewage outlets: *PAHs, Absorbable organic halogens (AOX), Aniline*	Four times/year on a seasonal basis (March, May, August, October)	
	2010–2013	Sediments, mariculture, and multiplication organisms: DDTs, HCHs, PCBs, chloramphenicol, sulfonamides for red tide disaster prevention and reduction	Once/year in August	

Note: the italicized fonts in the table, such as *DDTs*, *PCBs*, are selected items; Monitoring months are chosen based on the season in the Northern Hemisphere.

**Table 2:** Marine monitoring items including POPs in typical coastal provinces during “the 12th Five-Year” Plan of China.

Area	Year	Monitoring Projects and Indicators	Monitoring Frequency and Time	Remarks
South China Sea	2012–2015	Hazardous chemicals: tracking monitoring the status and changes of hazardous chemicals in seawater, sediments, marine organism in contaminated sea areas and adjacent areas, and monitoring the marine functional areas near hazardous chemical pollution incidents to understand the impact of hazardous chemical spills. Determine the specific time and frequency according to the amount and extent of the leakage of hazardous chemicals.		
	2014–2015	Pilot projects for carrying capacity of marine resources and environment: Distributions of pollutant source, river inflow, POPs discharged into the sea, pesticides and other prohibited substances	Once/year	Daya Bay
	2015	Water, sediments, seashells: PAHs in offshore oil exploration development	Once/year	
Hainan Province	2010–2012	Inshore mussel: HCHs, DDTs, PCBs	Once/year during August-October	
	2010–2013, 2015	Sediments: HCHs, DDTs, PCBs	Once/year in August	
	2010–2015	Sediments and biomass in mariculture zone: DDTs, PCBs	Once/year in August	
	2010–2013	Sediments, mariculture and multiplication organisms: DDTs, HCHs, PCBs, chloramphenicol, sulfonamides in red tide area	Sediments: once/year in August mariculture and multiplication organisms twice/year in May and August	
	2012–2014	Sewage toxic and hazardous substances in key sewage outlets into the sea: *choose 3–5 kinds of substances prohibited in international conventions*; General sewage outlets: *choose 1–2 kinds of characteristic pollutant*; Adjacent seawater, sediments: PAHs, OCPs, PCBs, phthalate, Bisphenol A, Nonylphenol and octylphenol.	Four times/year in March, May, August, October Water quality: twice/year; the remains: once/year	
	2013–2015	Hazardous chemicals: Identify monitoring items based on possible effects of hazardous chemicals on water quality, sediments and organisms	Determine the specific time and frequency according to the size and extent of the hazardous chemicals discharged	
Shandong Province	2013–2015	Sediments: PAHs, PCBs, *DDTs, HCHs, Phthalate esters (diethyl ester, dibutyl ester, diethylhexyl ester), Phenolic compounds (nonylphenol, octylphenol, bisphenol A)*	/	
	2013–2014	Sediments: DDTs, PCBs; Biomass of culture and enhancement-DDTs, PCBs, HCHs, Chloramphenicol, sulfonamides content in red tide area	Once/year in August	
	2013–2015	Sea water-PAHs, PCBs, carry out targeted monitoring of characteristic pollutants generated during the construction and operation phases of various marine (sea-related) projects in marine engineering construction project	Seawater-One monitoring during construction period and one monitoring during operation period	Large-scale reclamation activity area in Weifang Binhai New City
	2013–2015	Sewage toxic and hazardous substances in key sewage outlets into the sea: choose 3–5 kinds of substances prohibited by international conventions; General sewage outlets: *choose 1–2 kinds of characteristic pollutant*;	Four times/year in March, May, August, October	Identify monitoring items based on the main categories of seawater discharge
	2013–2015	River of entering the sea: *organophosphorus, pesticides, organochlorine pesticides, PAHs, PCBs, other environmental endocrine disruptors*	Monitoring frequency not less than three times/year, at least once each during low tide period, High tide period and slack water period	Monitoring projects can be appropriately increased based on the status of pollution sources in river basins
	2013–2014	Sediment and Biomass-DDTs, PCBs, Antibiotics Seawater aquaculture area	Once/year in August	
	2013–2015	Hazardous chemical pollution: Emergency monitoring and ecological damage assessment in coastal waters		
	2014	Sea water-*PAHs, DDTs, PCBs, Antibiotics* in aquaculture area	Four times/year in March, May, August, October	
	2015	Sea water: HCHs, DDTs, PCBs, PAHs	Sites for sediment monitoring need to be tested simultaneously	
Fujian Province	2012–2013, 2015	Sediments: DDTs, PCBs	Once/year in August	
	2012	Water, sediments: HCHs, DDTs, PCBs in key bay and island offshore water environment	Once/year in August	Fourteen bays along the coast of Fujian Province
	2012–2015	Inshore mussel: HCHs, DDTs, PCBs	Once/year in August	
	2012	Water, sediments: *PAHs*, Benzene in marine environmental emergencies monitoring		Selected items after 2012 are subject to availability
	2012–2015	General sewage outlet to sea: 2–3 species of characteristic pollutants Sea area adjacent to key sewage outlets: Sediment, Biomass-HCHs, DDTs, PCBs	Four times/year in March, May, August, October Once/year in August	Determine monitoring items based on the main categories of seawater discharge
	2012–2015	Sediments and cultured organisms; DDTs, PCBs, HCHs in Marine aquaculture zones		
	2013–2015	Biomass: DDTs, PCBs, HCHs in red tide area	Once/year in August	
	2014	Sediments-HCHs, DDTs, PCBs in marine dumping site	No less than Once/year	Moreover, according to the characteristics of the tipping area, the selected indexes can be added
	2015	Sediments: HCHs, DDTs, PCBs in mudflat	Once between the spring tide during July, August, and September.	

Note: the italicized fonts in the table, such as *DDTs PCBs*, are selected items; Monitoring months are chosen based on the season in the Northern Hemisphere.

## Supplementary Materials

The following are available online at https://www.mdpi.com/1660-4601/16/17/3083/s1, Table S1: Classification of the survey respondents; Questionnaire on POPs monitoring in the marine environment.
