# Supplementary material for: Current Status, Challenges, and Policy Recommendations of China’s Marine Monitoring Systems for Coastal Persistent Organic Pollution Based on Experts’ Questionnaire Analysis
Source: Int J Environ Res Public Health. 2019 Aug 25;16(17):3083. doi: 10.3390/ijerph16173083 (PMC6747340; doi:10.3390/ijerph16173083)
Supplement: Supplementary file 1 [file ijerph-16-03083-s001.pdf]

Supplementary Materials

Current Status, Challenges, and Policy Recommendations of China's Marine Monitoring Systems for Coastal Persistent Organic Pollution Based on Experts' Questionnaire Analysis

Wenlu Zhao [1,2,3], Huorong Chen [4], Jun Wang [5], Mingyu Zhang [3], Kai Chen [2,3,6], Yali Guo [3,6], Hongwei Ke [3], Wenyi Huang [3,7], Lihua Liu [8], Shengyun Yang [3,*] and Minggang Cai [2,3,6,*]

1 School of Environmental Science and Engineering, Zhejiang Gongshang University, Hangzhou 310018, China
2 Fujian Provincial Key Laboratory for Coastal Ecology and Environmental Studies, Xiamen University, Xiamen 361102, China
3 College of Ocean and Earth Science, Xiamen University, Xiamen 361102, China
4 Monitoring Center of Marine Environment and Fisheries Resources of Fujian, Fuzhou 350003, China
5 Department of Biological Technology, Xiamen Ocean Vocational College, Xiamen 361102, China
6 Coastal and Ocean Management Institute, Xiamen University, Xiamen 361102, China
7 East Sea Marine Environmental Investigating and Surveying Center, Ministry of Natural Resources, Shanghai 200137, China
8 Guangzhou Institute of Energy Conversion, Chinese Academy of Sciences, Guangzhou 510640, China
* Correspondence: yangsy@xmu.edu.cn (S.Y.), mgcai@xmu.edu.cn (M.C.)

Received: 21 July 2019; Accepted: 22 August 2019; Published: date

Table S1. Classification of the survey respondents.

Age			Gender		Education		
<30	30–40	>40	Male	Female	Bachelor	Master	PhD
18%	70%	12%	58%	40%	28%	48%	22%

Questionnaire on POPs monitoring in marine environment

1. Basic information:

**The name**		**gender**		**age**		**Education background/degree**	
**Work units**						**Position/title**	
**contact**							

Project introduction: in order to further protect offshore Marine environment in China, the research of persistent organic pollutants (POPs) management status, promote the offshore Marine environment pollution prevention and control work, needs to understand the current POPs monitoring work in offshore environment, through the surveillance staff questionnaire investigation of the project, his concern and timely sum up, organized the event, please participate.

2. Monitoring questionnaire survey (please thicken or color the options red)

(1) the general situation

1. Which of the following tasks are you most concerned about in the business development of China's environmental monitoring system?

A. To strengthen the monitoring of pollutant flux into the sea and its spatial and temporal distribution in the Marine environment.

B. We will strengthen integrated monitoring with ecological health monitoring and monitoring of various Marine functional areas as the core.

C. Realize the monitoring data and information sharing, improve the monitoring product deep processing ability.

D. Enhance the service awareness of Marine environmental monitoring agencies and improve their service efficiency.

E. We will innovate and optimize technologies for monitoring the Marine environment and develop and improve the system for Marine environmental assessment.

F. We will strengthen the building of the talent pool and build a professional and innovative talent pool.

G. We will improve the distribution of the Marine environmental monitoring system and promote the capacity building of local Marine environmental monitoring institutions.

H. We will actively participate in regional and international Marine environmental monitoring and assessment projects and properly address global environmental issues.

2. Which of the following areas of environmental monitoring are you most interested in :(choose 3-5)

A. The determination of environmental priority control pollutant list in China.

B. Monitoring of ecological diseases and new types of pollutants.

C. Monitoring of non-point source pollution of Marine environment.

D. Comprehensive monitoring of large-scale Marine ecological environment.

E. On-site, real-time and online monitoring of key sewage discharge areas, ecological sensitive areas and red tide monitoring areas.

F. Airborne remote sensing monitoring.

G. Red tide and oil spill warning.

H. Responsibility identification and loss assessment for monitoring pollution incidents.

I. Other.

3. In your opinion, the main reasons influencing the improvement of China's Marine environmental monitoring technology are :(choose 3-5 items)

A. The existing standard methods and technical procedures are backward, which is not conducive to the introduction of advanced monitoring technology.

B. Marine environmental monitoring institutions have a thin base and are unable to learn and disseminate advanced monitoring technologies.

C. The traditional monitoring technology has been able to meet the existing task requirements, so there is no need to adopt more advanced monitoring technology for the time being.

D. The distribution and resource allocation of Marine environmental monitoring forces need to be further optimized.

E. China's science and technology development environment is relatively backward.

F. Lack of innovation awareness.

G. Other.

(b) awareness of POPs

4. How do you know POPs?

A. professional knowledge

B. more familiar

C. general knowledge
D. no understanding

5. What characteristics of POPs do you know? (multiple choice)
A. high toxicity
B. persistence
C. bioaccumulation
D. long-distance migration

6. What do you think are the hazards of POPs? (multiple choice)
A. There are carcinogenic, teratogenic and mutagenic hazards to human body
B. It can be amplified step by step through the food chain, causing high toxicity harm to organisms and human beings
C. causing neurological and immune disorders in the organism
D. no harm

7. Which of the following POPs do you think belong to? (multiple choice)
A. pesticides
B. polychlorinated biphenyls (PCBs)
C. dioxins and furan
D. polycyclic aromatic hydrocarbons (PAHs) e. mercury (Hg)

8. What are the main sources of POPs in your opinion? (pops)
A. use of pesticides
B. use of industrial chemicals
C. by-products in commercial or industrial production

9. How well do you know about POPs monitoring and assessment in the international Marine environment?
A. Basic understanding of international development status of POPs
B. Basic understanding of POPs monitoring and evaluation in Marine environments in developed countries
C. Basic knowledge of POPs monitoring and evaluation projects in major regional Marine environments undertaken by international organizations
D. Basic knowledge of POPs in global Marine environment and related international cooperation projects
E. POPs monitoring and assessment in the international Marine environment are not of concern

POPs monitoring in offshore Marine environments

10. Do you think there is a need for regular or regular monitoring of POPs?
A. yes
B. no
C. no

11. In your opinion, which aspects of your current monitoring work should include POPs monitoring?
A. Routine monitoring of water quality
B. monitoring of sediment and biomass
C. monitoring of oil spill pollution d. others

12. Your operational evaluation of POPs monitoring:
    A. strong
    B. general
    C. poor

13. Difficulty of POPs monitoring in your opinion:
    A. big
    B. medium
    C. small

14. In your opinion, the difficulty of POPs monitoring is (multiple choice) :
    A. the condition of pollution is not clear
    B. sampling method
    C. factor analysis
    D. evaluation
    E. others

15. How reliable do you think the POPs monitoring data is?
    A. high
    B. high
    C. general
    D. low
16. If the reliability is considered low, the main reason is (multiple choices):
    A. representativeness of samples
    B. analytical equipment
    C. evaluation methods and standards
    D. others

17. Choice of POPs monitoring items:
    A. moderate
    B. too much
    C. too little

18. POPs monitoring items that you think need attention:
    A. POPs in the priority control category for the performance of our country in international conventions
    B. Monitoring of new types of pollutants
    C. is not clear

19. In your opinion, the water body monitoring frequency of POPs should be one year:
    A. once, August
    B. Three times in May, August and October
    C. six times, two months or one month
    D. once a month
    E. monitoring in case of emergency

20. In your opinion, the frequency of sediment and biological monitoring of POPs should be one year:
    A. once, August
    B. Three times in May, August and October
    C. six times, two months or one month
    D. once a month
    E. monitoring in case of emergency

21. In your opinion, the atmospheric monitoring frequency of POPs should be one year:
    A. once, August
    B. Three times in May, August and October
    C. six times, two months or one month
    D. once a month
    E. monitoring in case of emergency

22. In POPs monitoring, you rank the importance of media monitoring as:
    A. Water quality > sediment quality = biological monitoring > atmosphere
    B. Water quality monitoring > sediment quality monitoring > biological monitoring > atmosphere
    C. water quality = sediment quality > biological monitoring > atmosphere
    D. Water quality = sediment quality = biological monitoring > atmosphere
    E. water quality = sediment quality = biological monitoring = atmosphere
    F. other
    Please explain why you chose:

23. Where you think POPs monitor stations should be:
    A. Consistent with the layout of conventional water quality monitoring stations
    B. Set only at specific outlets or contaminated areas
    C. is not clear

24. What are the current sampling methods of POPs monitoring in your unit?

25. Are you willing to undertake POPs monitoring and analysis?
    A. is
    B. no

If the answer is: (A) In your opinion, the most pressing technical problem to be solved in the next step of work is:

A. Early improvement of unified technical procedures or industry standards
B. Technical training on relevant sampling and analysis methods
C. Participate in interlab and internal quality control
D. Development of standard solutions or reference materials
E. Other

If the answer is: (B) no, the main reason is:

A. Lack of necessary analytical testing equipment
B. Lack of proper pre-processing equipment
C. Technical personnel lack relevant experience and ability
D. Analytical instruments are overburdened, overburdened, and have no time
E. other

(4) the analytical and measurement capacity of POPs samples, and the evaluation of the processing and sharing system of the result data

26. Whether the monitoring capacity of your unit matches the POPs sample collection task (multiple choices)
    A. The POPs samples can be collected well with the basic matching
    B. Technical force is not enough, sample collection and analysis task is difficult
    C. Lack of sampling equipment, unable to collect all samples as required
    D. It is difficult to further carry out POPs monitoring task because of insufficient personnel allocation

E. Other

27. Do you think there is a problem of backward technical means in the process of POPs monitoring data collection, management and analysis?
    A exists and
    B doesn't

28. Your existing organic pollutant monitoring capabilities include
    A. Volatile organic pollutants
    B. Polycyclic aromatic hydrocarbons,
    C. Organochlorine pesticide
    D. Bromo flame retardant
    E. Polychlorinated biphenyls (PCBS)
    F. phthalate
    G. phenolics
    H. Organophosphorus pesticide
    I. antibiotic
    J. other

29. In your opinion, the main gap between the evaluation method of POPs monitoring results and actual demand in China's offshore environment is :(choose 3-5 items)
    A. The evaluation method system is not perfect, and POPs can not be evaluated scientifically, objectively, quickly and comprehensively
    B. The future pollution trend of POPs in offshore environment cannot be predicted scientifically and accurately
    C. The regional characteristics of the Marine environment have not been fully taken into account by adopting unified national evaluation standards
    D. The status and trend evaluation of POPs in offshore environment are mostly in the qualitative stage, and the source and contribution of POPs cannot be directly determined
    E. The POPs capacity of the national and regional Marine environment has not been recognized
    F. There is a lack of mature methods for evaluating the ecological toxicology or ecological health damage caused by POPs
    G. There is a lack of POPs for offshore Marine ecological risk assessment aimed at protecting human health
    H. Other

30. The main defects of POPs in China's existing standard system for offshore Marine environmental monitoring are :(multiple choices)
    A. The technical methods and equipment used in the standard methods are backward
    B. Too little POPs were covered and the content was not updated in a timely manner
    C. The POPs concentration baseline in accordance with the characteristics of China's offshore Marine environment has not been established, resulting in the setting of standard parameters not in line with the actual situation
    D. The use of standard substances needs to be further standardized and institutionalized
    E. Other

31. What are your views on POPs monitoring data and information sharing in your organization or peers (multiple choice)
    A. It can be Shared among all Marine environmental monitoring agencies
    B. Data and information on Marine environmental monitoring can be Shared regionally
    C. Monitoring data and information can be Shared with other Marine and environmental authorities

D. The data and information of Marine environmental monitoring from different sources are very different. The quality of data is difficult to be guaranteed and it is difficult to share

E. The Marine environmental monitoring information system is not perfect and it is difficult to share

F. Other

32. How do you see the applicability of POPs to monitoring of pollution in the current Marine environmental monitoring technical regulations?
    A. The technical details are clearly described and operable
    B. Some areas have been described as ominous but not POPs affecting monitoring
    C. some technical details are not clear
    D. there are obvious deficiencies and difficult practical operation

Monitoring personnel and training mechanisms

33. How about POPs training in your organization?
    A often
    B occasionally
    C doesn't

34. Please select the most suitable technical training method in your opinion :(multiple choices are available)
    A. Training courses shall be uniformly organized by relevant national monitoring departments
    B. Training courses will be held by the Marine area monitoring center, and the national center will send technicians to participate in the guidance
    C. Training courses shall be organized by provincial monitoring institutions, and technical personnel from the national center and the sea area center shall participate in the guidance
    D. Technical learning shall be conducted by the monitoring unit itself, such as inviting professionals from scientific research institutions to give lectures, or studying abroad for exchanges, etc
    E. Field sampling instruction
    F. Other

35. How is your participation in international cooperation and exchanges involving POPs Marine environmental monitoring (multiple choice)
    A. Host or participate in global international cooperation projects
    B. To host or participate in regional international cooperation projects
    C. Participate in international seminars on Marine environmental monitoring and assessment
    D. Visit and exchange with foreign Marine environmental monitoring institutions
    E. Attend lectures, lectures or technical training of foreign experts
    F. Never participated in international cooperation activities or projects

(6) quality control and assurance

36. Do you think the current quality control and assurance system of our unit can meet the needs of POPs monitoring and guarantee work?
    A.Adapt and meet
    B. basic adapt and meet
    C. not adapt

37. If not, what are the main problems (multiple choices)?

A. The quality assurance system of monitoring institutions has not been established and the quality management work has not been implemented properly;

B. The environmental conditions of the laboratory cannot meet the monitoring needs, and the laboratory infrastructure is rudimentary;

C. The monitoring personnel are not properly allocated and the establishment cannot meet the work requirements;

D. There is a gap between the operational quality of personnel and the requirements for monitoring, and there is a lack of professional and technical personnel and experienced monitors;

E. others, please specify the specific content:

38. What do you think of the Marine environment monitoring in remobilised sample collection, storage and transportation field testing, samples, sample preparation, analysis, testing, data processing, data transmission and so on in the whole process of quality control is the most important and the most easy to appear quality problem is which of the following links (can choose more)?
    A. Sample collection;
    B. Field test;
    C. Storage and transportation of samples;
    D. Sample preparation and analysis testing;
    E. Data processing and transmission;
    F. In addition, please specify the specific content:

39. What do you think are the following (multiple options) aspects of POPs data quality management and quality control in Marine environmental monitoring?
    A. External control sample assessment;
    B. Laboratory capacity verification;
    C. Sample comparison test;
    D. Quality supervision and inspection;
    E. Evaluation of assessment results;
    F. In addition, please specify the specific content:

40. We would like to hear your comments and Suggestions on how to improve the effectiveness of Marine environmental monitoring quality assurance, or what relevant measures and countermeasures should be adopted.
